# Long noncoding RNASEH1‐AS1 exacerbates the progression of non‐small cell lung cancer by acting as a ceRNA to regulate microRNA‐516a‐5p/FOXK1 and thereby activating the Wnt/β‐catenin signaling pathway

**DOI:** 10.1002/cam4.4509

**Published:** 2022-02-15

**Authors:** Chan Zhang, Jian Huang, Ke Lou, Hui Ouyang

**Affiliations:** ^1^ Department of Respiratory Medicine The Fourth Hospital of Changsha Changsha Hunan China

**Keywords:** ceRNA network, epithelial‐mesenchymal transition, RNASEH1 antisense RNA 1, Wnt/β‐catenin pathway

## Abstract

**Background:**

Till now, no study has focused on the functions of RNASEH1 antisense RNA 1 (RNASEH1‐AS1) in non‐small cell lung cancer (NSCLC). Accordingly, we measured the expression of RNASEH1‐AS1 in NSCLC and characterized its functions in detail. Finally, our research elucidated the mechanisms that occurred downstream of RNASEH1‐AS1.

**Methods:**

RNASEH1‐AS1 expression was examined utilizing TCGA database and qRT‐PCR. Functional experiments were conducted to study the tumor‐associated functions of RNASEH1‐AS1. The targeting relationship among RNASEH1‐AS1, microRNA‐516a‐5p (miR‐516a‐5p), and forkhead box K1 (FOXK1) was revealed utilizing RNA immunoprecipitation and luciferase reporter assays.

**Results:**

Utilizing TCGA database and our own cohort, we found a significantly increased level of RNASEH1‐AS1 in NSCLC. The high level of RNASEH1‐AS1 was markedly related with poor clinical outcomes. Knockdown of RNASEH1‐AS1 expression inhibited NSCLC cell growth, metastatic capacities, and epithelial‐mesenchymal transition and promoted the apoptosis in vitro, whereas RNASEH1‐AS1 overexpression exerted the opposite effects. Additionally, knocking down RNASEH1‐AS1 expression suppressed tumor growth in vivo. RNASEH1‐AS1 was confirmed to act as a miR‐516a‐5p sponge, consequently upregulating FOXK1 expression in NSCLC cells. As revealed by the subsequent rescue experiments, the miR‐516a‐5p/FOXK1 axis served as a downstream effector of RNASEH1‐AS1. In addition, by controlling the miR‐516a‐5p/FOXK1 axis, RNASEH1‐AS1 was capable of activating the Wnt/β‐catenin pathway.

**Conclusion:**

RNASEH1‐AS1 exacerbated the oncogenicity of NSCLC by affecting the miR‐516a‐5p/FOXK1 axis and consequently promoting the activation of Wnt/β‐catenin pathway. Our newly identified RNASEH1‐AS1/miR‐516a‐5p/FOXK1/Wnt/β‐catenin network may offer an interesting foundation for NSCLC treatment in the clinic.

## Graphical abstract

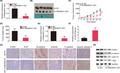

## 1 INTRODUCTION

Lung cancer is the most frequently diagnosed cancer and the leading cause of tumor‐associated death around the world.^1^ Moreover, the worldwide incidence and mortality rates of lung cancer have continued to increase for many years.^2^ More than one‐third of new NSCLC cases are diagnosed in China, and this rate of diagnosis causes a notably heavy public health burden.^3^ Lung cancer includes two different histological subtypes: non‐small cell lung cancer (NSCLC) and small cell lung cancer; the former responsible for approximately 80%–85% of all lung cancer cases.^4^ In clinical practice, only a relatively small number of NSCLC patients is diagnosed in the early stage and are eligible to undergo surgical excision. If it is not detected at an early stage, the disease can progress to a middle or late stage with local or distant metastasis, and the best opportunity for treatment is lost.^5^ Despite the substantial progress in the development of therapeutic strategies in recent decades, patients with NSCLC often experience poor clinical outcomes.^6^ Therefore, to improve therapeutic efficiency, it is necessary to reveal the molecular events that occur during NSCLC pathogenesis, which are currently largely unknown.

Long noncoding RNAs (lncRNAs) are a class of RNA molecules that are 200 nucleotides in length and lack protein‐coding capacity.^7^ Previously, lncRNAs were considered to be useless byproducts of genome transcription; however, an increasing amount of evidence has shown the important roles of lncRNAs in physiological and pathological biological processes.^8^, ^9^, ^10^ Recently, increasing numbers of studies have revealed the dysregulation of lncRNA expression in almost all types of human cancer, and their aberrant expression is required for inducing malignancy and tumorigenesis.^11^ In NSCLC, lncRNAs play cancer suppressive or carcinogenic roles and thus affect the onset and progression of NSCLC.^12^

MicroRNAs (miRNAs) are a family of endogenous, single‐stranded, and noncoding RNA molecules that consist of approximately 17–25 nucleotides.^13^ By complementary interacting with the 3′‐UTRs of downstream targets, miRNAs decrease gene expression via translation attenuation or mRNA degradation.^14^ MiRNAs are closely associated with NSCLC pathogenesis, and they perform crucial actions in controlling tumor‐related gene expression, thus regulating the aggressive phenotypes of NSCLC.^15^ The competitive endogenous RNA (ceRNA) theory has been described and used to construct a key regulatory pathway through which lncRNAs can adsorb specific miRNAs and thereby modify the levels of downstream target mRNAs.^16^

Untill now, no study has illustrated the expression and functions of RNASEH1 antisense RNA 1 (RNASEH1‐AS1) in NSCLC. Our research revealed that RNASEH1‐AS1 is overexpressed in NSCLC. Additionally, functional experiments were conducted to identify its tumor‐related roles.

## 2 MATERIALS AND METHODS

### 2.1 Subjects

This research was approved by the Research Ethics Committee of The Fourth Hospital of Changsha. We collected NSCLC tissues from 59 patients in our hospital, and all of these subjects provided written informed consent. None anticancer therapies were administered to these participants. All fresh tissues were preserved in liquid nitrogen. The exclusion criteria were: patients who had been treated with anticancer therapies, patients with other types of human cancer, and patients with severe liver and kidney dysfunction.

### 2.2 Cell lines

Three NSCLC cell lines, namely, the H460, H522, and H1299 cell lines, were maintained in PMI 1640 medium (Gibco; Thermo Fisher Scientific, Inc.). The NSCLC cell lines A549 and SK‐MES‐1 were, respectively, grown in F‐12K and MEM medium. BEAS‐2B cell line was cultured in bronchial epithelial cell growth medium. Furthermore, 10% fetal bovine serum (FBS) was added to all the cell culture media. All the aforementioned cell lines (ATCC) were grown in a cell incubator at 37°C in 5% CO_2_.

### 2.3 Transfection

The NSCLC cells were transfected with a miR‐516a‐5p mimic or inhibitor (GenePharma Co., Ltd), and cell lines in which miR‐516a‐5p was overexpressed or downregulated were obtained. The miRNA negative control (NC) mimic and NC inhibitor were used for comparison. Specific siRNAs targeting RNASEH1‐AS1 (si‐RNASEH1‐AS1), FOXK1 (si‐FOXK1), and NC siRNA (si‐NC) were also acquired from GenePharma. The si‐RNASEH1‐AS1#1 sequence was 5′‐CCCAATTGATCTAGTAGTAAAGT‐3′; the si‐ RNASEH1‐AS1#2 sequence was 5′‐GTGAAGAAGATG AAATGTTTAGC‐3′; and the si‐NC sequence was 5′‐CACGATAAGACAATGTATTT‐3′. GenScript Biotech Corp. provided pcDNA3.1‐FOXK1 and pcDNA3.1‐RNASEH1‐AS1. Transient transfection was performed with Lipofectamine^®^ 2000 (Invitrogen). Subsequent to 6 h of cultivation, complete culture medium was added to replace the transfection reagent.

### 2.4 Quantitative real‐time polymerase chain reaction (qRT‐PCR)

The extraction of total RNA was achieved utilizing TRIzol Universal Reagent (Tiangen). After quantification via a Nanodrop2000 (OD260) (Thermo Fisher Scientific), detection of miR‐516a‐5p was implemented applying miRcute miRNA First‐Strand cDNA Synthesis Kit and miRcute miRNA qPCR Detection Kit SYBR Green (both from Tiangen). U6 served as the reference gene for normalization. For the measurement of RNASEH1‐AS1 and FOXK1 levels, PrimeScript reagent Kit was utilized for the reverse transcription reaction, while quantitative PCR was conducted with the help of PrimeScript™ RT Master Mix (both from Takara). RNASEH1‐AS1 and FOXK1 levels were normalized to GAPDH expression. Gene expression was calculated with the 2^−ΔΔCq^ method.^17^ The sequences of all primers are presented in Table 1.

**TABLE 1:** Primer sequences used for qRT‐PCR

Gene	Sequence (5′–3′)
RNASEH1‐AS1	Forward: GCGGATCTACAGTAAGGGCTGT
	Reverse: CGCCCTCCTTTGTGCTTATTC
FOXK1	Forward: CACTGTACCCCCAGATCTCCC
	Reverse: CGTTCTGCACAAAGCGGTAA
GAPDH	Forward: CGGAGTCAACGGATTTGGTCGTAT
	Reverse: AGCCTTCTCCATGGTGGTGAAGAC
miR‐218‐5p	Forward: TCGGCAGGUUGUGCUUGAUC
	Reverse: CACTCAACTGGTGTCGTGGA
miR‐516a‐5p	Forward: TCGGCAGGUUCUCGAGGAAA
	Reverse: CACTCAACTGGTGTCGTGGA
U6	Forward: GCTTCGGCAGCACATATACTAAAAT
	Reverse: CGCTTCACGAATTTGCGTGTCAT

### 2.5 Subcellular fractionation experiment

The segregation of the nuclear and cytoplasmic fractions was realized with a Cytoplasmic and Nuclear RNA Purification Kit (Norgen). After RNA extraction, the expression distribution of RNASEH1‐AS1 was investigated utilizing qRT‐PCR.

### 2.6 Fluorescence in situ hybridization

The in situ localization of RNASEH1‐AS1 was proven with the Fluorescent In Situ Hybridization Kit (RiboBio). After receiving the fixation, utilizing 4% paraformaldehyde, NSCLC cells were incubated with probes specifically targeting RNASEH1‐AS1 at 37°C overnight. The whole process was performed in the dark. Immediately thereafter, DNA was stained with Hoechst solution. Finally, a confocal laser scanning microscope (Leica) was used for imaging.

### 2.7 Cell counting kit (CCK)‐8 assay

We seeded 100 μl of cell suspensions harboring 2 × 10^3^ transfected cells into 96‐well plates. Cell proliferation was monitored daily until Day 3. After being cultured with 10 μl CCK‐8 reagent (Beyotime) at 37°C for 2 h, the optical density at 450 nm wavelength (OD450) was measured with a microplate reader.

### 2.8 Colony formation assay

The 6‐well plates were seeded with 500 transfected cells suspended. The cells were grown at 37°C for 2 weeks to form colonies. We replaced the culture medium every 3 days. Finally, 100% methanol was used to fix the colonies with over 50 cells, which were then stained with 0.1% crystal violet. The colonies were captured with an inverted microscope (Olympus Corp.).

### 2.9 Flow cytometry analysis

Transfected cells were harvested with trypsin and collected by centrifugation. We then resuspended the cells in 195 μl Annexin V‐FITC binding buffer, which was came from an Annexin V‐FITC Apoptosis Detection Kit (Beyotime). Totally 5 µl Annexin V‐FITC and 10 µl PI was added into cell suspension. Cell apoptosis was examined with a flow cytometer (BD Biosciences).

### 2.10 Transwell migration and invasion experiments

We harvested transfected cells at 48 h post transfection and resuspended them in FBS‐free medium. For the cell migration experiment, we added 200 μl of cell suspensions containing 1 × 10^4^ cells to the upper chambers of Transwell inserts (BD Biosciences). The lower compartments were filled with complete culture medium. Twenty‐four hours later, 0.1% crystal violet solution was used to stain the migrated cells. After extensive washing, the numbers of migrated cells were counted under a light microscope (×200 magnification). For the cell invasion experiment, the Transwell inserts were pre‐coated with Matrigel, and the experimental procedures followed that of the migration experiment.

### 2.11 Tumor xenograft experiments

The in vivo tumor xenograft experiments were implemented under the permission from the Animal Care and Use Committee of the Fourth Hospital of Changsha. To develop a stable RNASEH1‐AS1 depletion cell line, shRNA specific for RNASEH1‐AS1 (sh‐RNASEH1‐AS1), and NC shRNA (sh‐NC) were cloned into a lentiviral vector (GenePharma Co., Ltd). The sh‐RNASEH1‐AS1 sequence was 5′‐CCGGCCCAATTGATCTAGTAGTAAAGTCTCGAGACTTTACTACTAGATCAATTGGGTTTTTG‐3′, and the sh‐NC sequence was 5′‐CCGGCACGATAAGACAATGTATTTCTCGAGAAATACATTGTCTTATCGTGTTTTTG‐3′. SK‐MES‐1 cells were transfected with lentiviral vectors, and cells with stable RNASEH1‐AS1 knockdown were selected with puromycin.

Four‐week‐old BALB/c nude mice (HFK Bioscience) were subcutaneously inoculated with 2 × 10^6^ SK‐MES‐1 cells stably overexpressing sh‐RNASEH1‐AS1. Each group contained five nude mice, and all mice were housed under specific pathogen‐free conditions at 25°C and 50% humidity, with a 10:14 light/dark cycle and ad libitum access to food and water. After 1 week, the sizes of the xenografts were measured every 3 days. Tumor volumes were analyzed using the following formula: volume = length × width^2^/2. Thirty‐one days later, the mice were euthanized, and the tumor xenografts were removed for weighing and immunohistochemistry.

### 2.12 Bioinformatics prediction

The binding between RNASEH1‐AS1 and miR‐516a‐5p was predicted utilizing StarBase 3.0 (http://starbase.sysu.edu.cn/). A publicly available algorithm, namely, TargetScan (http://www.targetscan.org), was employed for searching the downstream target of miR‐516a‐5p.

Kaplan–Meier Plotter (https://kmplot.com/analysis/) was utilized to unveil the association between RNASEH1‐AS1 level and overall survival of patients with NSCLC.

### 2.13 Luciferase reporter assay

The wild‐type (wt) or mutant (mut) RNASEH1‐AS1 fragments synthesized by GenePharma were inserted into the psiCHECK™‐2 vector (Promega), generating the RNASEH1‐AS1‐wt and RNASEH1‐AS1‐mut reporter plasmids. Concurrently, FOXK1‐wt and FOXK1‐mut reporter plasmids were designed and produced. The wt or mut plasmid alongside the miR‐516a‐5p/NC mimic was transfected into NSCLC cells. The luciferase activity of the samples was investigated with a Dual‐Luciferase^®^ Reporter Assay System (Promega).

### 2.14 Western blotting

RIPA lysis buffer and an enhanced BCA protein assay kit (Beyotime) were applied for protein isolation and quantification, respectively. SDS–PAGE was used for total protein separation, which were then transferred onto PVDF membranes. The membranes were subsequently cultivated with primary antibodies targeting FOXK1 (sc‐373810; Santa Cruz Biotechnology, Inc.), E‐cadherin (ab212059), N‐cadherin (ab76011), Vimentin (ab92547), β‐catenin (ab265591), cyclin D1 (ab16663), and c‐Myc (ab32072) or GAPDH (ab128915; all from Abcam). After an overnight incubation at 4°C, horseradish peroxidase‐conjugated secondary antibodies were adopted to incubate the membranes. The protein bands were visualized with a Pierce™ TMB Substrate Kit (Thermo Fisher Scientific).

### 2.15 RNA immunoprecipitation

The RNA immunoprecipitation assay was achieved applying an EZ‐Magna RIP™ RNA Binding Protein Immunoprecipitation Kit (Millipore). NSCLC cells were lysed by incubation with complete RIP buffer. Next, RIP buffer supplemented with magnetic beads conjugated to anti‐Ago2 or normal mouse IgG (Millipore) was incubated with the cell lysates overnight at 4°C. After rinsing with RIP washing buffer, the proteins were digested via Proteinase K buffer treatment. The immunoprecipitated RNA was detected by qRT‐PCR.

### 2.16 Statistical analysis

All the experiments were repeated three times, and the obtained data presented as mean ±standard deviation were analyzed using SPSS software 19.0 (SPSS). Student's *t*‐test and one‐way analysis of variance with Tukey's test were applied for comparing data between groups. Pearson's correlation coefficient was used to assess gene expression relation. *p* < 0.05 indicated statistical significance.

## 3 RESULTS

### 3.1 RNASEH1‐AS1 exacerbates the malignant biological phenotype of NSCLC cells

**FIGURE 1:**
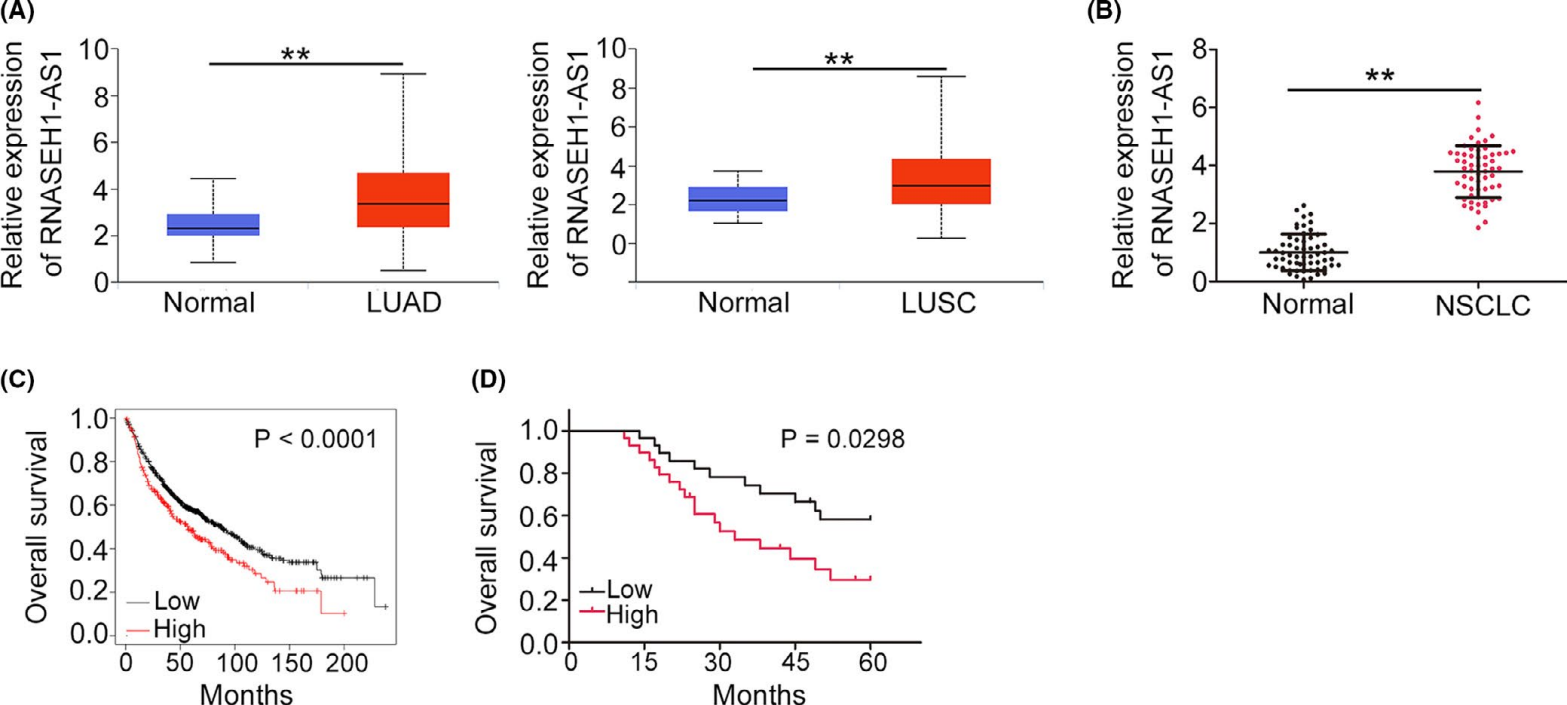
High RNASEH1‐AS1 expression is verified in NSCLC. (A) RNASEH1‐AS1 level in LUAD and LUSC tissues was analyzed utilizing TCGA. (B) qRT‐PCR was used to determine the RNASEH1‐AS1 levels in NSCLC tissues. (C) Kaplan–Meier Plotter was utilized to analyze the relationship between RNASEH1‐AS1 level and overall survival in patients with LUAD and LUSC. (D)The survival of NSCLC patients in our cohort with high‐ or low RNASEH1‐AS1 level. **p<0.01

First, we conducted comparative analyses with the TCGA database and investigated the expression of RNASEH1‐AS1 in lung adenocarcinoma (LUAD) and lung squamous cell carcinoma (LUSC). LUAD and LUSC tissues clearly overexpressed RNASEH1‐AS1 (Figure 1A). Additionally, NSCLC tissues from our own cohort exhibited enhanced expression of RNASEH1‐AS1 (Figure 1B). According to the median value of RNASEH1‐AS1, all patients with NSCLC were divided into low‐ or high‐RNASEH1‐AS1 expression groups. Using Kaplan–Meier Plotter (https://kmplot.com/analysis/), we found that NSCLC patients with high RNASEH1‐AS1 level displayed shorter overall survival in contrast to patients with low RNASEH1‐AS1 level (Figure 1C), which is consistent with the Kaplan–Meier survival analysis from our cohort (Figure 1D).

**FIGURE 2:**
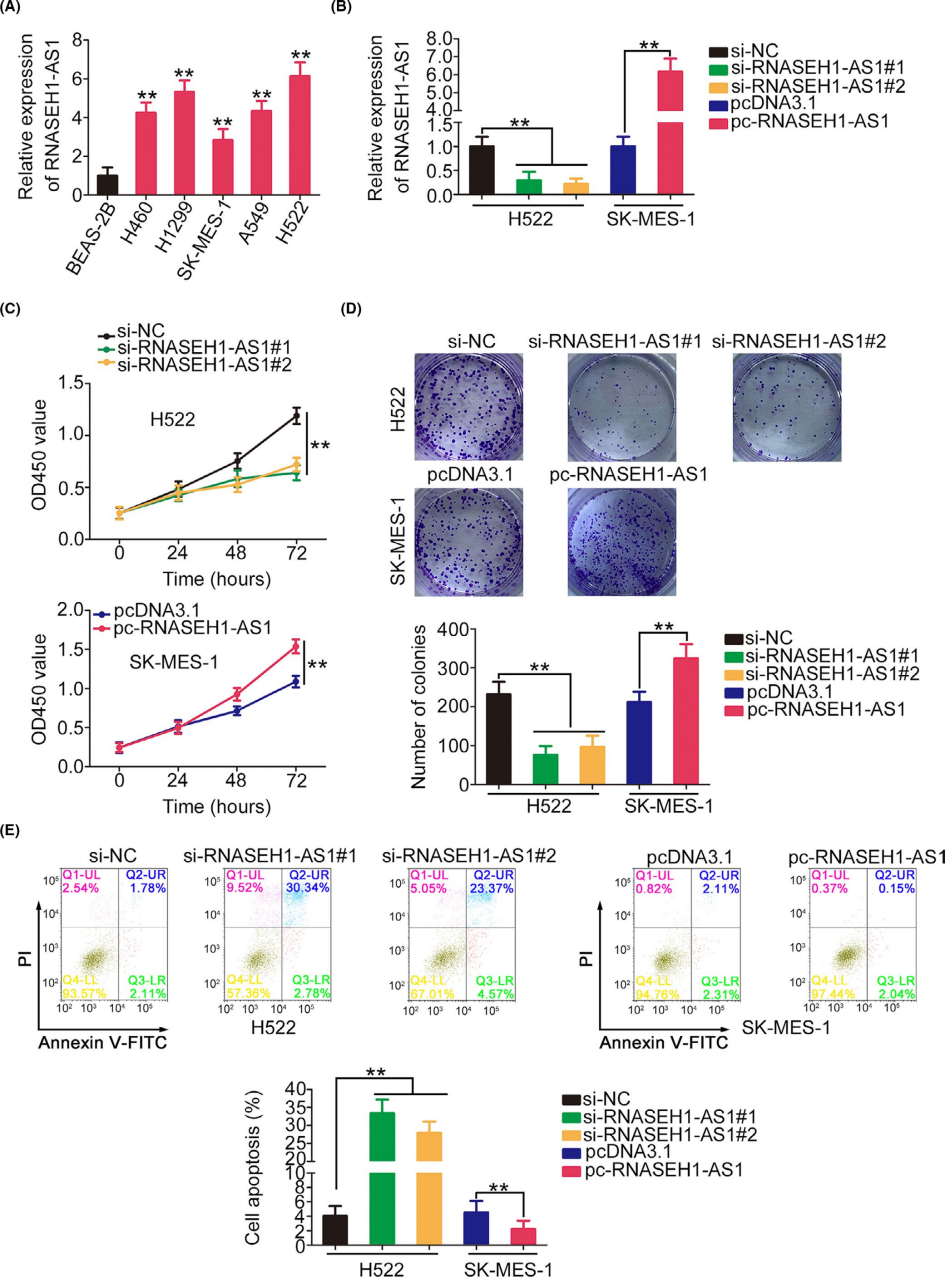
RNASEH1‐AS1 regulates NSCLC cell progression. (A) NSCLC cell lines were subjected to qRT‐PCR to assess RNASEH1‐AS1 expression. (B) The RNASEH1‐AS1 levels in si‐RNASEH1‐AS1‐transfected or pc‐RNASEH1‐AS1‐transfected cells were examined. (C, D) The proliferation of NSCLC cells with si‐RNASEH1‐AS1 or pc‐RNASEH1‐AS1 transfection. (E) Cell apoptosis was assessed in NSCLC cells in which RNASEH1‐AS1 was knocked down or overexpressed. **p<0.01

Before we explored the specific functions of RNASEH1‐AS1, RNASEH1‐AS1 level in NSCLC cell lines was measured, and the upregulation of RNASEH1‐AS1 in all the tested NSCLC cell lines was observed (Figure 2A). The H522 cell line was used for loss‐of‐function experiments because it expressed the highest levels of RNASEH1‐AS1 of the five NSCLC cell lines. To prevent off‐target effects, two siRNAs, namely, si‐RNASEH1‐AS1#1 and si‐RNASEH1‐AS1#2, were used to knock down RNASEH1‐AS1. Moreover, SK‐MES‐1 cells, which expressed the lowest levels of RNASEH1‐AS1, were treated with pcDNA3.1‐RNASEH1‐AS1 for gain‐of‐function experiments (Figure 2B). The proliferation and colony‐forming capacities were severely impaired after RNASEH1‐AS1 knockdown but enhanced after RNASEH1‐AS1 overexpression (Figure 2C,D). RNASEH1‐AS1‐knockdown H522 cells exhibited obvious increase in apoptosis compared with si‐NC‐transfected cells. Similarly, the apoptosis rate was notably decreased in SK‐MES‐1 cells overexpressing RNASEH1‐AS1 (Figure 2E).

**FIGURE 3:**
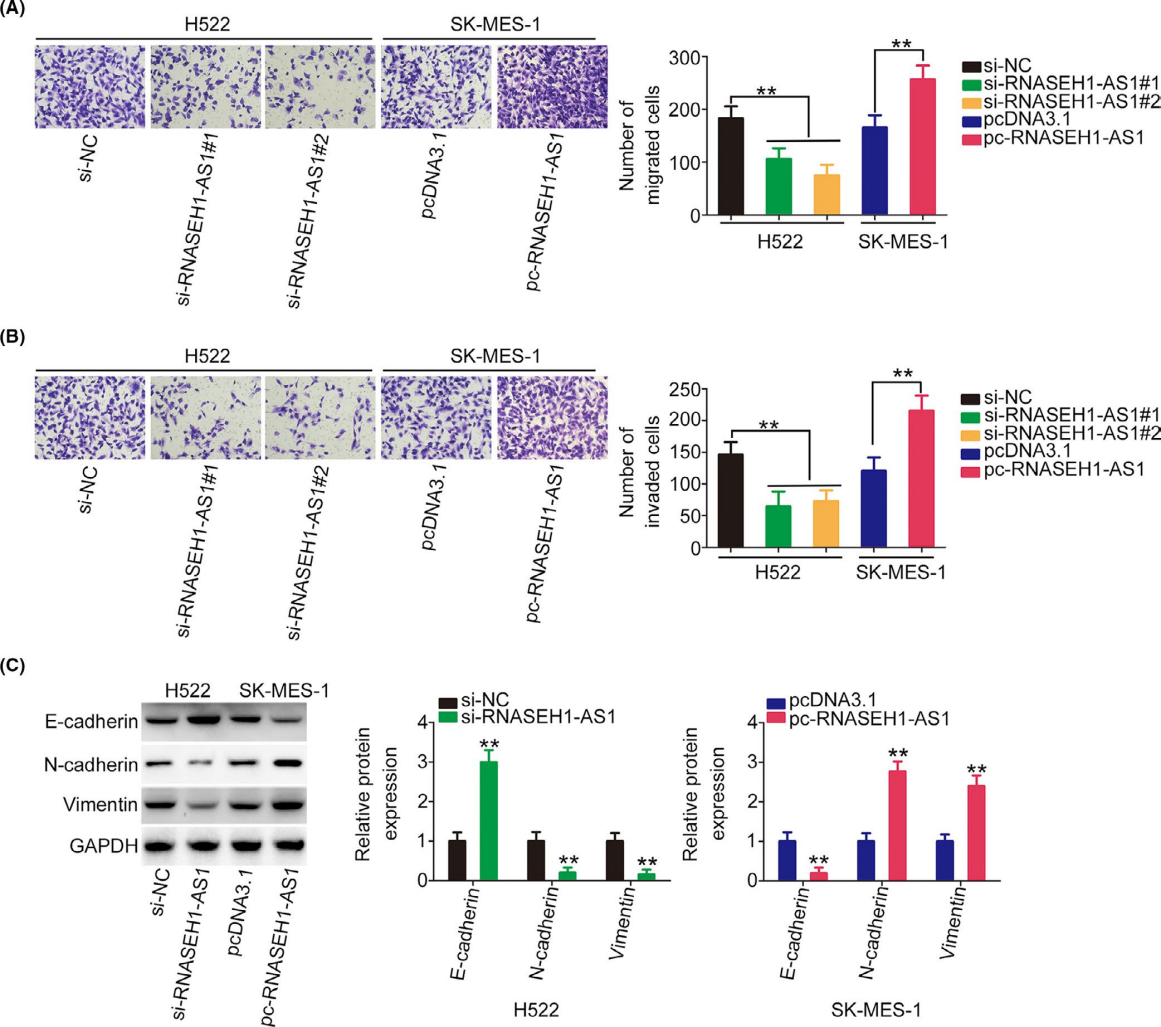
RNASEH1‐AS1 affects NSCLC cell metastasis and ETM. (A, B) NSCLC cell motility after the downregulation or upregulation of RNASEH1‐AS1 expression (×200 magnification). (C) The expression of N‐cadherin, Vimentin, and E‐cadherin in NSCLC cells after the downregulation or upregulation of RNASEH1‐AS1. **p<0.01

The regulatory effect of RNASEH1‐AS1 on metastasis was investigated. The knockdown of RNASEH1‐AS1 expression decreased the motility of the NSCLC cells, while pcDNA3.1‐RNASEH1‐AS1 treatment yielded the opposite result (Figure 3A,B). Furthermore, the western blotting results verified that RNASEH1‐AS1 knockdown decreased N‐cadherin and Vimentin expression while increasing E‐cadherin level in H522 cells. In contrast, N‐cadherin and Vimentin levels were increased, whereas E‐cadherin level was decreased, in the pcDNA3.1‐RNASEH1‐AS1‐transfected SK‐MES‐1 cells (Figure 3C). Overall, RNASEH1‐AS1 exerted carcinogenic effects in NSCLC cells.

### 3.2 RNASEH1‐AS1 serves as a miR‐516a‐5p sponge

**FIGURE 4:**
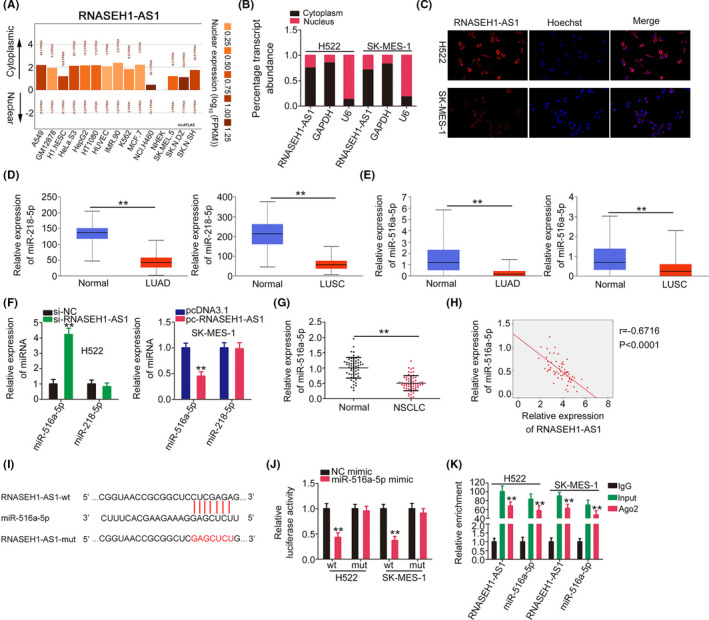
miR‐516a‐5p is sponged by RNASEH1‐AS1. (A) The subcellular localization of RNASEH1‐AS1 predicted by LncATLAS. (B, C) The subcellular location of RNASEH1‐AS1 in NSCLC cells. (D, E) Expression profiles of miR‐218‐5p and miR‐516a‐5p in LUAD and LUSC tissues were determined utilizing the TCGA database. (F) The expression of miR‐218‐5p and miR‐516a‐5p was examined in RNASEH1‐AS1‐knockdown or RNASEH1‐AS1‐overexpressing NSCLC cells. (G) MiR‐516a‐5p expression in NSCLC. (H) The relationship between miR‐516a‐5p and RNASEH1‐AS1 levels in NSCLC tissues. (I)The wt and mut binding sequences between RNASEH1‐AS1 and miR‐516a‐5p. (J) The luciferase activity of RNASEH1‐AS1‐wt or RNASEH1‐AS1‐mut in miR‐516a‐5p‐overexpressing NSCLC cells. (K) RIP experiment corroborated the interaction between RNASEH1‐AS1 and miR‐516a‐5p. **p<0.01

To reveal the molecular events downstream of RNASEH1‐AS1, we first used LncATLAS (http://lncatlas.crg.eu/) to predict the subcellular location of RNASEH1‐AS1. RNASEH1‐AS1 was predicted to be mostly distributed in cell cytoplasm (Figure 4A), which was reconfirmed by subcellular fractionation experiments (Figure 4B) and fluorescence in situ hybridization (Figure 4C). This observation suggests that RNASEH1‐AS1 may perform its tumor‐promoting functions by acting as a ceRNA. According to StarBase 3.0, RNASEH1‐AS1 was predicted to contain possible binding sites for a total of 15 miRNAs (Table 2). Utilizing TCGA, miR‐218‐5p (Figure 4D) and miR‐516a‐5p (Figure 4E) were revealed to be downregulated in LUAD and LUSC tissues; thus, they were chosen for experimental verification. Then, the levels of the two candidates were measured in RNASEH1‐AS1‐knockdown or pcDNA3.1‐RNASEH1‐AS1‐transfected NSCLC cells. Transfection with si‐RNASEH1‐AS1 or pcDNA3.1‐RNASEH1‐AS1 caused a significant increase or decrease in miR‐516a‐5p expression, respectively, whereas miR‐218‐5p level was unchanged in response to changes in RNASEH1‐AS1 expression (Figure 4F). Besides, miR‐516a‐5p was underexpressed in NSCLC tissues and displayed an inverse relationship with RNASEH1‐AS1 level (Figure 4G,H).

**TABLE 2:** Potential miRNAs that may be sequestered by RNASEH1‐AS1 were predicted by starBase 3.0

miRNA name	miRNA name
miR‐1306‐5p	miR‐516a‐5p
miR‐1323	miR‐548o‐3p
miR‐151a‐5p	miR‐5691
miR‐151b	miR‐576‐5p
miR‐218‐5p	miR‐579‐5p
miR‐324‐5p	miR‐6805‐3p
miR‐345‐5p	miR‐942‐5p
miR‐409‐5p	

Luciferase reporter assay was then conducted to further verify the targeting site between RNASEH1‐AS1 and miR‐516a‐5p (Figure 4I). MiR‐516a‐5p overexpression hindered the luciferase activity of RNASEH1‐AS1‐wt; however, when the targeting sequences were mutated, this inhibitory effect on the luciferase activity was abrogated (Figure 4J).

Ago2 is an important element of RNA‐induced silencing complex (RISC) and required for miRNA‐mediated gene silencing. It initiates the degradation of target genes by means of its catalytic activity in gene silencing processes guided by miRNAs. To address whether RNASEH1‐AS1 relates with RISC complex, RIP assay was performed, and we measured RNASEH1‐AS1 and miR‐516a‐5p in the Ago2 immunoprecipitates. RNASEH1‐AS1 and miR‐516a‐5p were evidently enriched in the Ago2 immunoprecipitates (Figure 4K). Thus, miR‐516a‐5p was sequestered by RNASEH1‐AS1.

### 3.3 RNASEH1‐AS1 acts as a ceRNA to regulate the miR‐516a‐5p/FOXK1 axis

**FIGURE 5:**
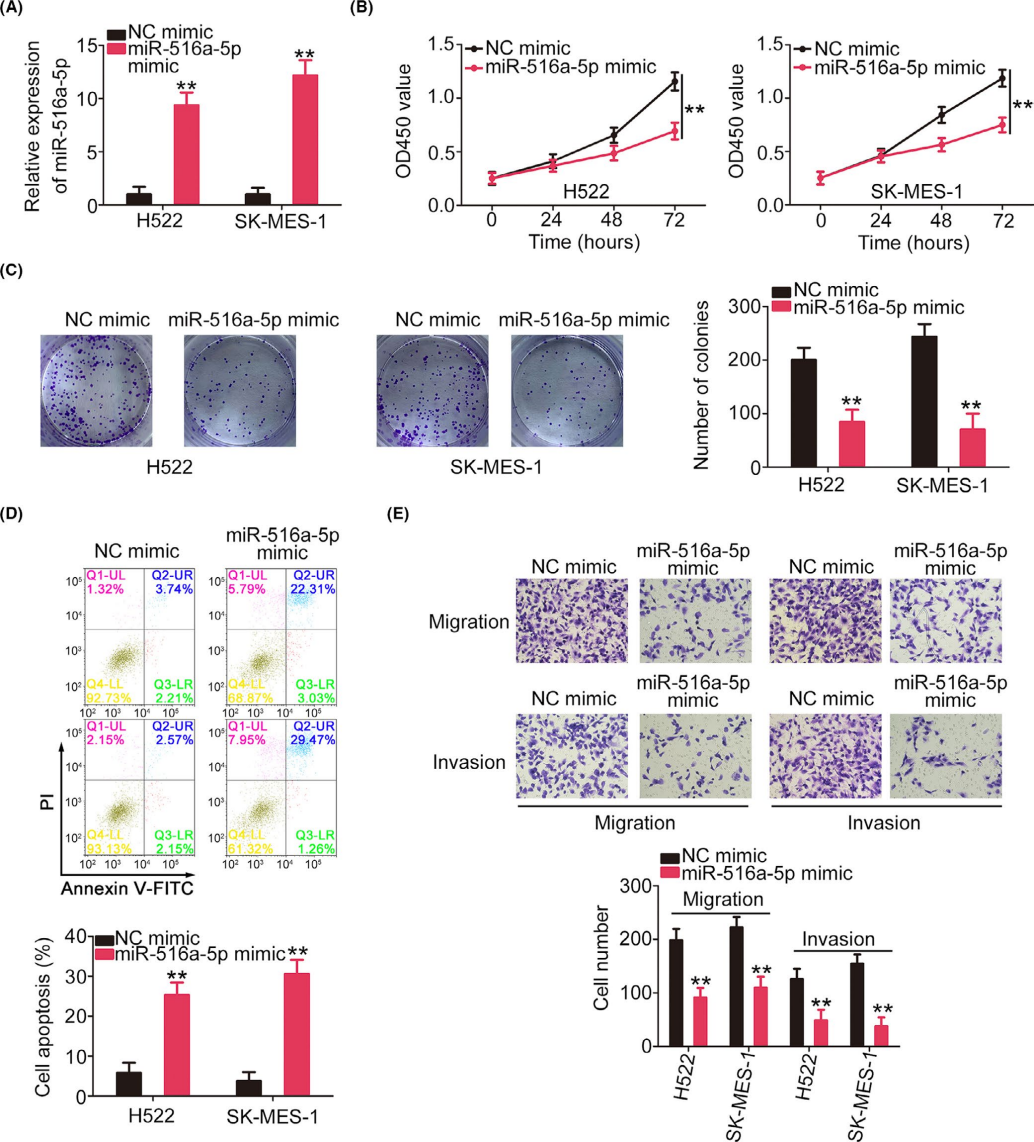
MiR‐516a‐5p is an anti‐oncogenic miRNA in NSCLC. (A) The miR‐516a‐5p expression in miR‐516a‐5p mimic‐transfected NSCLC cells. (B, C) NSCLC cell proliferation after miR‐516a‐5p overexpression. (D) The apoptosis of miR‐516a‐5p‐overexpressed NSCLC cells. (E) The motility of miR‐516a‐5p‐overexpressed NSCLC cells was investigated (×200 magnification). **p<0.01

A miR‐516a‐5p upregulation model was successfully established in NSCLC cells by miR‐516a‐5p mimic transfection (Figure 5A). Ectopic miR‐516a‐5p expression led to decreased cell proliferation and colony‐forming (Figure 5B,C), which were accompanied by enhanced cell apoptosis (Figure 5D). Additionally, the motility of NSCLC cells was hindered by the miR‐516a‐5p mimic (Figure 5E).

**FIGURE 6:**
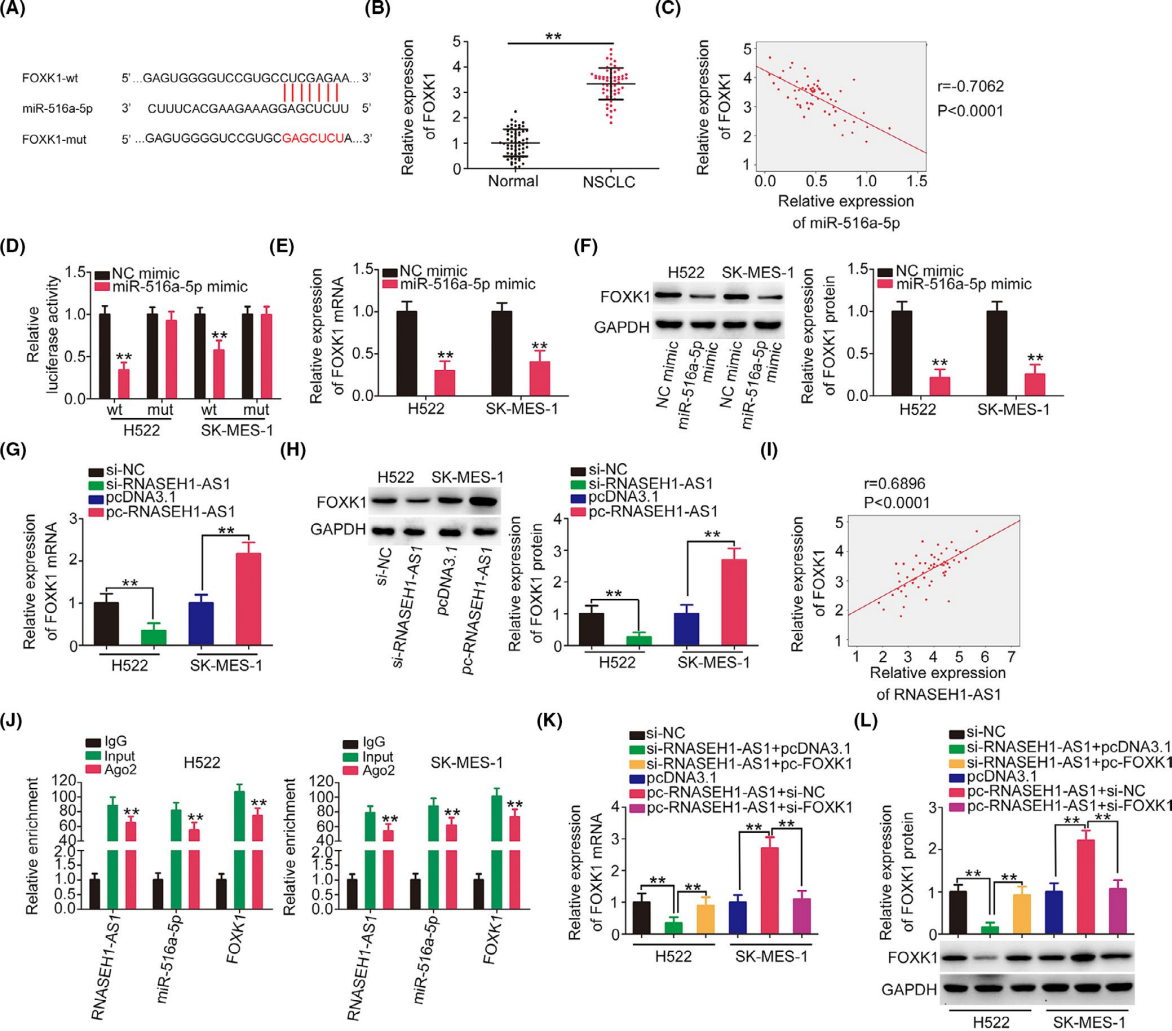
FOXK1 is regulated by the RNASEH1‐AS1/miR‐516a‐5p axis. (A) The wt and mut sequences between miR‐516a‐5p and FOXK1 are shown. (B) FOXK1 level in NSCLC. (C) The expression relationship between miR‐516a‐5p and FOXK1 in NSCLC tissues. (D) The luciferase activity of NSCLC cells after cotransfection with FOXK1‐wt or FOXK1‐mut and the miR‐516a‐5p mimic or NC mimic was detected. (E, F) FOXK1 expression was measured in miR‐516a‐5p‐overexpressing NSCLC cells. (G, H) RNASEH1‐AS1‐knockdown or RNASEH1‐AS1‐overexpressing NSCLC cells were subjected to the assessment of FOXK1 expression. (I) The expression relationship between FOXK1 and RNASEH1‐AS1 in NSCLC tissues. (J) RIP experiment verified the interaction among RNASEH1‐AS1, miR‐516a‐5p, and FOXK1. (K, L) si‐RNASEH1‐AS1‐transfected or pc‐RNASEH1‐AS1‐transfected NSCLC cells were treated with the miR‐516a‐5p inhibitor or the miR‐516a‐5p mimic. Then, FOXK1 expression was examined in the different groups. **p<0.01

Then, we used bioinformatics tool to find potential targets of miR‐516a‐5p. FOXK1 harbored possible binding sequences for miR‐516a‐5p (Figure 6A) and was thoroughly investigated due to its important oncogenic roles in NSCLC. FOXK1 expression was verified to be overexpressed in NSCLC tissues, and a negative relationship was affirmed between FOXK1 expression and miR‐516a‐5p levels (Figure 6B,C). The miR‐516a‐5p‐overexpressing NSCLC cells exhibited decreased luciferase activity triggered by FOXK1‐wt, but not of FOXK1‐mut (Figure 6D). In addition, miR‐516a‐5p mimic‐transfected NSCLC cells exhibited decreased FOXK1 expression levels (Figure 6E,F).

Mechanistic studies were implemented to reveal the relationship among RNASEH1‐AS1, miR‐516a‐5p, and FOXK1. Knockdown of RNASEH1‐AS1 decreased the expression of FOXK1; in contrast, transfection with pc‐RNASEH1‐AS1 notably increased the expression of FOXK1 (Figure 6G,H). A positive relationship between RNASEH1‐AS1 and FOXK1 levels was also demonstrated in our own cohort (Figure 6I). A notably increasing enrichment of RNASEH1‐AS1, miR‐516a‐5p, and FOXK1 was observed in Ago2 immunoprecipitates (Figure 6J). Through rescue assays, loss of RNASEH1‐AS1 expression caused a decrease in FOXK1 level in H522 cells, which was mostly recovered by miR‐516a‐5p inhibitor cotransfection. Moreover, treatment with miR‐516a‐5p mimic neutralized the impacts of pc‐RNASEH1‐AS1 on FOXK1 expression (Figure 6K,L). In summary, RNASEH1‐AS1 sponged miR‐516a‐5p and thereby affected the FOXK1 levels.

### 3.4 RNASEH1‐AS1 attenuates the aggressive processes of NSCLC cells by regulating the miR‐516a‐5p/FOXK1 axis

**FIGURE 7:**
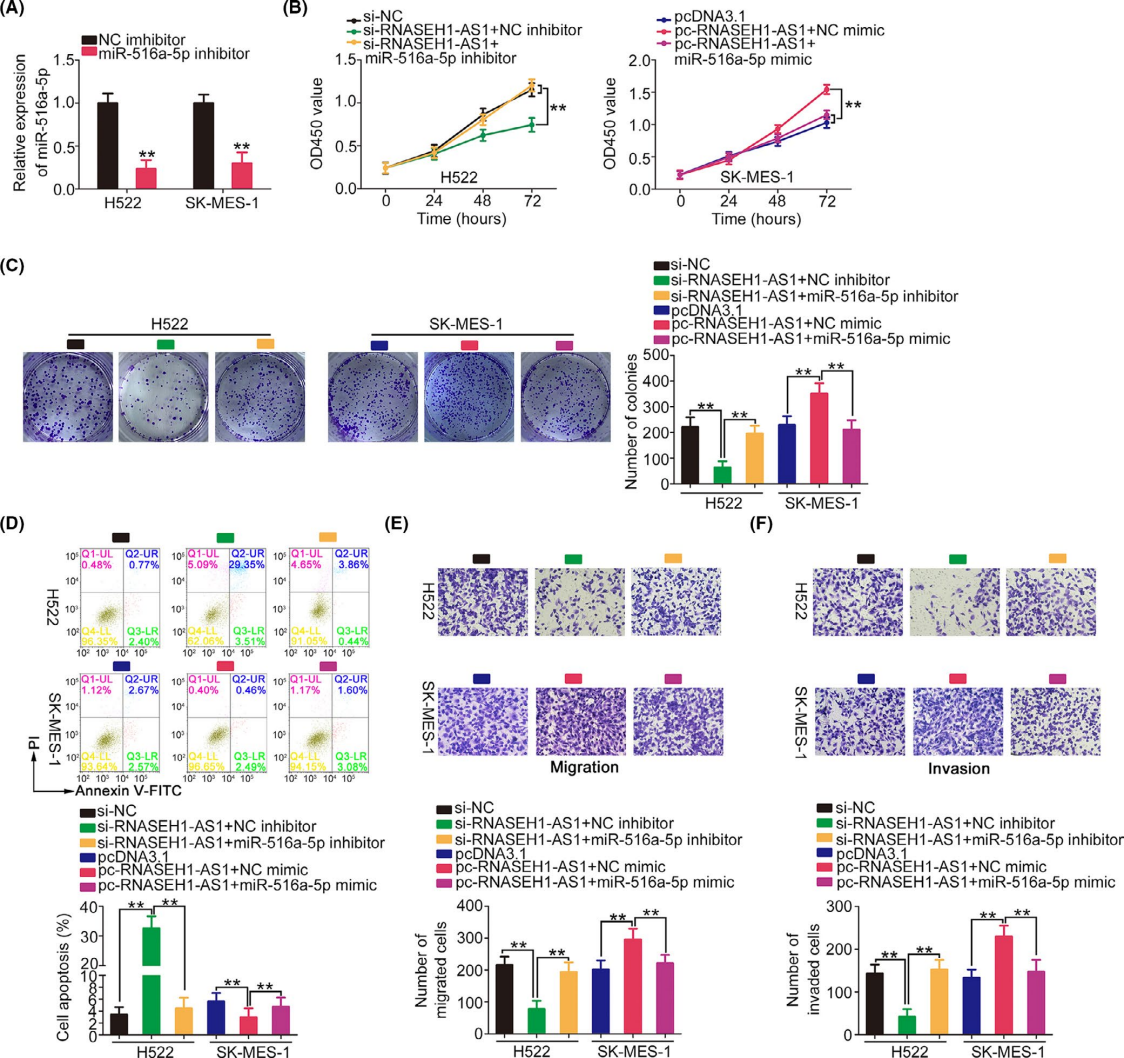
Regulation of malignant characteristics of NSCLC cells by RNASEH1‐AS1 is dependent on miR‐516a‐5p. (A) MiR‐516a‐5p level in miR‐516a‐5p inhibitor‐transfected NSCLC cells. (B–D) si‐RNASEH1‐AS1 alongside the miR‐516a‐5p/NC inhibitor was transfected into H522 cells, while SK‐MES‐1 cells overexpressing RNASEH1‐AS1 were transfected with miR‐516a‐5p/NC mimic. Cell growth and apoptosis were assessed in the different groups. (E, F) The motility of the abovementioned cells was examined (×200 magnification). **p<0.01

**FIGURE 8:**
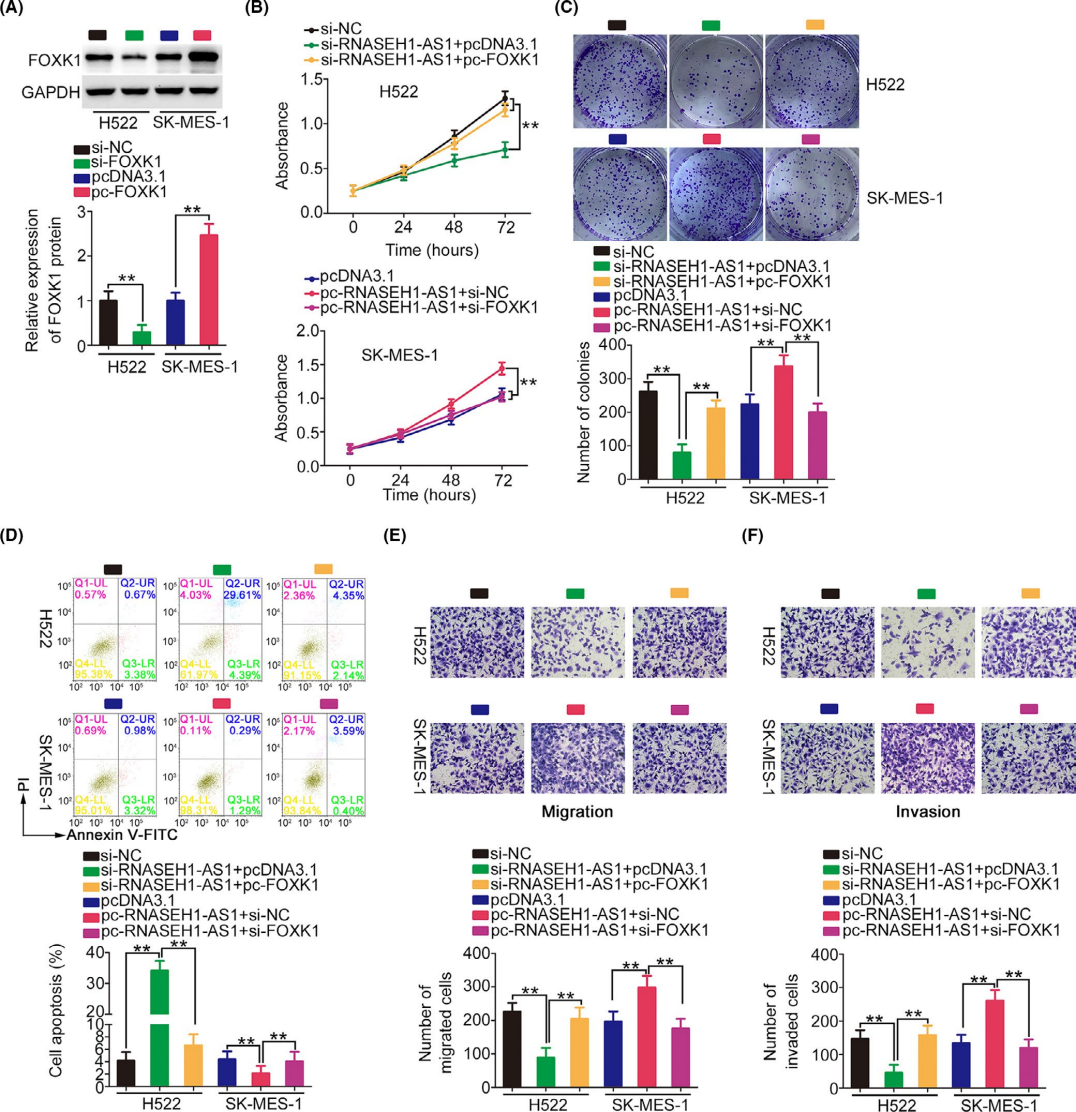
FOXK1 can counteract the role of RNASEH1‐AS1. (A) The FOXK1 protein levels were successfully increased and decreased in NSCLC cells after pcDNA3.1‐ FOXK1 and si‐FOXK1 treatment, respectively. (B–F) RNASEH1‐AS1‐deficient H522 cells were treated with pcDNA3.1 or pcDNA3.1‐ FOXK1, while SK‐MES‐1 cells were treated with pcDNA3.1, pcDNA3.1‐RNASEH1‐AS1+si‐NC, or pcDNA3.1‐RNASEH1‐AS1+si‐FOXK1. A series of experiments were implemented to assess cell functional change (×200 magnification). **p<0.01

Finally, whether the miR‐516a‐5p/FOXK1 axis was required for the regulatory effects of RNASEH1‐AS1 was determined with rescue experiments. First, the efficiency of the miR‐516a‐5p inhibitor was verified via qRT‐PCR (Figure 7A). NSCLC cell proliferation was inhibited by si‐RNASEH1‐AS1 but enhanced by pc‐RNASEH1‐AS1; however, the effects were abolished after miR‐516a‐5p knockdown and miR‐516a‐5p overexpression (Figure 7B,C). Additionally, the miR‐516a‐5p inhibition reversed the improvement in cell apoptosis caused by RNASEH1‐AS1 downregulation. Furthermore, miR‐516a‐5p mimic decreased the influence of pc‐RNASEH1‐AS1 on cell apoptosis (Figure 7D). The decreased migration and invasion of RNASEH1‐AS1‐knockdown NSCLC cells was recovered by miR‐516a‐5p inhibitor, whereas miR‐516a‐5p overexpression abrogated the effect of pc‐RNASEH1‐AS1 on promoting cell motility (Figure 7E,F).

pc‐FOXK1 and si‐FOXK1 were also used in rescue experiments, and western blotting confirmed the transfection efficiencies (Figure 8A). Reintroduction of FOXK1 reversed the regulatory effects of si‐RNASEH1‐AS1. In addition, the increased growth and motility as well as the decreased apoptosis of NSCLC cells overexpressing RNASEH1‐AS1 were reversed by FOXK1 knockdown (Figure 8B–F). In short, RNASEH1‐AS1 affected NSCLC malignancy by controlling miR‐516a‐5p/FOXK1 axis.

### 3.5 RNASEH1‐AS1 activates the Wnt/β‐catenin pathway by modulating miR‐516a‐5p/FOXK1 axis

**FIGURE 9:**
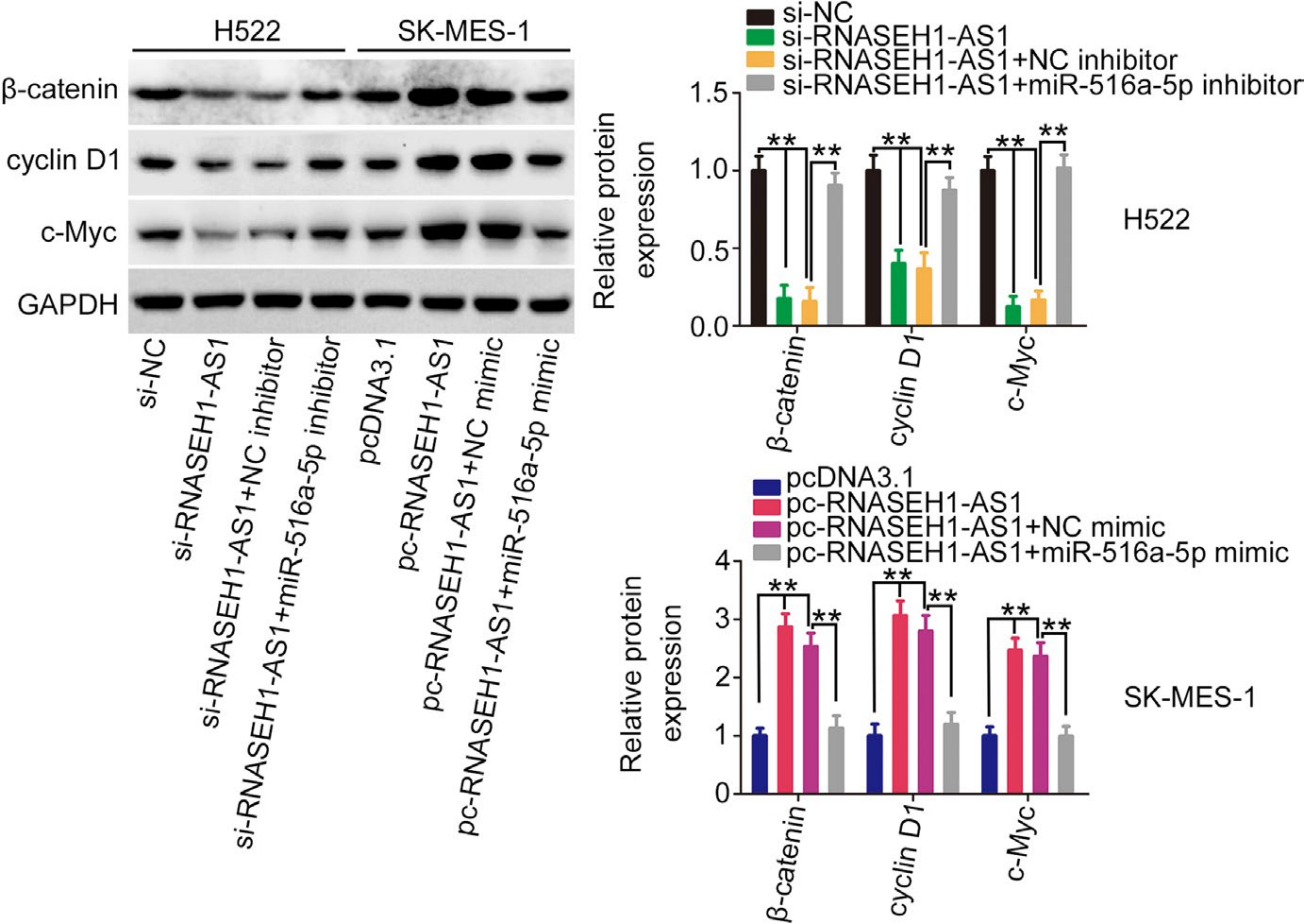
RNASEH1‐AS1 affects the Wnt/β‐catenin pathway. H522 cells were transfected with si‐NC, si‐RNASEH1‐AS1+NC inhibitor, or si‐RNASEH1‐AS1+ miR‐516a‐5p inhibitor. SK‐MES‐1 cells were transfected with pcDNA3.1, pc‐RNASEH1‐AS1+NC mimic, or pc‐RNASEH1‐AS1+ miR‐516a‐5p mimic. After transfection, expression levels of β‐catenin, cyclin D1 and c‐Myc were examined. **p<0.01

FOXK1 was proven to be an activator of the Wnt/β‐catenin pathway.^18^ ^,^ ^19^ Accordingly, we continued to investigate whether RNASEH1‐AS1 was involved in the regulation of the Wnt/β‐catenin signaling. The β‐catenin, cyclin D1, and c‐Myc levels were decreased by si‐RNASEH1‐AS1, whereas pc‐RNASEH1‐AS1 exerted opposite effects. However, the repressing action of si‐RNASEH1‐AS1 on these molecules was reversed by miR‐516a‐5p inhibitor treatment, while miR‐516a‐5p overexpression recovered the levels that had been increased in response to RNASEH1‐AS1 upregulation (Figure 9). Thus, RNASEH1‐AS1 enhanced the activation Wnt/β‐catenin pathway via the regulation of miR‐516a‐5p/FOXK1 axis.

### 3.6 Knocking down RNASEH1‐AS1 attenuates tumor growth in vivo

**FIGURE 10:**
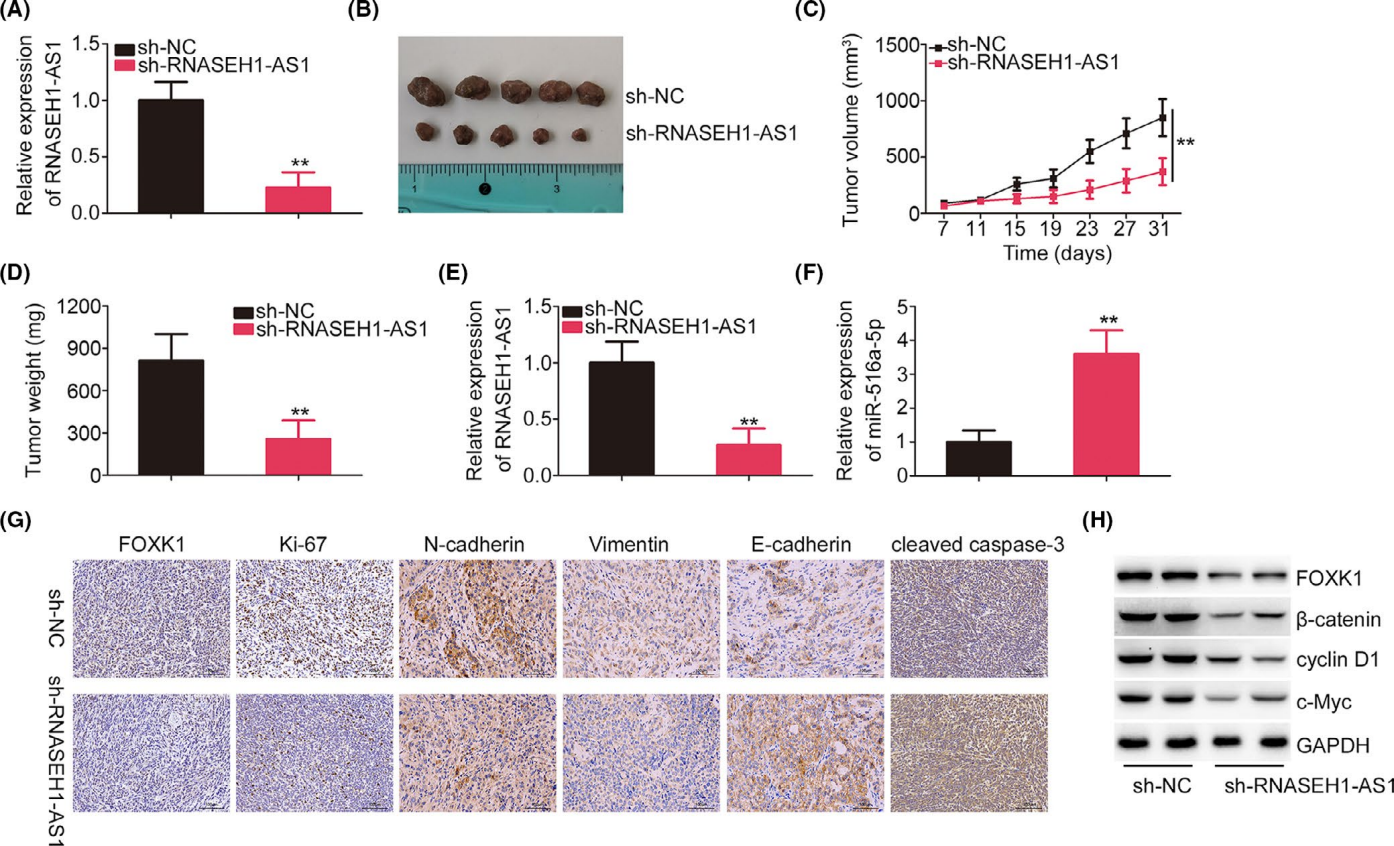
RNASEH1‐AS1 deficiency inhibits tumor growth in vivo. (A) Transfection efficiency of sh‐RNASEH1‐AS1. (B) Typical images of tumor xenografts are shown. (C) The growth curves of tumor xenografts. (D) The weight of tumor xenografts. (E, F) RNASEH1‐AS1 and miR‐516a‐5p expression in tumor xenografts. (G) Immunohistochemistry was used to analyze FOXK1, Ki‐67, N‐cadherin, Vimentin, E‐cadherin, and cleaved Caspase‐3 expression in tumor xenografts. (H) The protein levels of FOXK1, β‐catenin, cyclin D1, and c‐Myc in tumor xenografts. **p<0.01

RNASEH1‐AS1 expression was stably silenced in SK‐MES‐1 cells using shRNA lentivirus particles (Figure 10A). In contrast to the sh‐NC group, the RNASEH1‐AS1 knockdown group exhibited markedly reduced growth of tumor xenografts (Figure 10B,C). Additionally, the tumor xenograft weights in the sh‐RNASEH1‐AS1 group were obviously decreased (Figure 10D). Furthermore, molecular detection revealed that the RNASEH1‐AS1 level was decreased, while the miR‐516a‐5p was overexpressed, in tumor xenografts transfected with sh‐RNASEH1‐AS1 (Figure 10E,F). Moreover, immunohistochemistry analysis verified that FOXK1, Ki‐67, N‐cadherin, and Vimentin levels were decreased, whereas cleaved Caspase‐3 and E‐cadherin levels were increased in sh‐RNASEH1‐AS1 group (Figure 10G). Besides, the protein levels of FOXK1, β‐catenin, cyclin D1, and c‐Myc were downregulated in shRNASEH1‐AS1‐transfected tumor xenografts (Figure 10H). In summary, RNASEH1‐AS1 silencing inhibited tumor growth and epithelial‐mesenchymal transition (ETM) processes in vivo.

## 4 DISCUSSION

To date, substantial evidence has revealed that the onset and progression of NSCLC are affected not only by protein‐coding genes, but also by non‐protein‐coding genes.^20^, ^21^, ^22^ Thus, an intensive assessment of the detailed roles and working mechanisms of lncRNAs in NSCLC would be conducive to exploit promising theoretical evidence to develop targeted therapy. Many lncRNAs exist in the human genome; however, their roles in NSCLC pathogenesis are not completely understood. Herein, we determined the RNASEH1‐AS1 expression in NSCLC and characterized its clinical value. Concurrently, functional experiments were conducted to study the tumor‐associated roles of RNASEH1‐AS1. Ultimately, our research also revealed the mechanisms that occurred downstream of RNASEH1‐AS1.

A clear importance of lncRNAs in the oncogenicity of NSCLC has recently been verified. For instance, CCDC144NL‐AS1,^23^ AK027294,^24^ and HOXA11‐AS^25^ are overexpressed in NSCLC and exacerbate malignancy. In contrast, downregulation of ZNF674‐AS1,^26^ WT1‐AS,^27^ and MEG3^28^ is identified in NSCLC and can lower the aggressive events of NSCLC. However, the functions of RNASEH1‐AS1 in NSCLC have rarely been explored. Herein, utilizing TCGA database and our own cohort, we revealed a significant increase in the level of RNASEH1‐AS1 in NSCLC. RNASEH1‐AS1 upregulation was markedly related with poor clinical outcomes. RNASEH1‐AS1 knockdown inhibited the growth, metastatic capacities and ETM, and promoted the apoptosis of NSCLC cells in vitro, whereas RNASEH1‐AS1 overexpression exerted the opposite actions. Additionally, knocking down RNASEH1‐AS1 expression inhibited tumor growth and ETM in vivo. In short, these observations enhance the understanding of the complex mechanism that occur during NSCLC pathogenesis.

The deeper molecular events underlying the anticancer activities of RNASEH1‐AS1 knockdown are still unknown. To address this problem, the location of RNASEH1‐AS1 in NSCLC cells was initially examined because the working mechanisms of RNASEH1‐AS1 are likely determined by its subcellular distribution. Our data showed that most RNASEH1‐AS1 was located in the cytoplasm of NSCLC cells. Over the years, the ceRNA theory authenticated that cytosolic lncRNAs can sequester miRNAs and thereby alleviate the miRNA‐mediated decrease in target mRNA levels, consequently exerting regulatory effects.^29^ A bioinformatics tool revealed that miR‐516a‐5p contained complementary bases that might pair with RNASEH1‐AS1. Subsequently, RNASEH1‐AS1 was shown to act as a miR‐516a‐5p sponge. Furthermore, utilizing bioinformatics prediction, luciferase reporter assay and molecular detection, FOXK1 was certified as a downstream target of miR‐516a‐5p. FOXK1 expression was further revealed to be positively modulated by RNASEH1‐AS1, and the effect was realized by sponging miR‐516a‐5p. Altogether, three RNAs, namely, RNASEH1‐AS1, miR‐516a‐5p, and FOXK1, form a newly identified ceRNA pathway in NSCLC cells.

MiR‐516a‐5p exhibits different expression and functional patterns in different human cancers. For instance, miR‐516a‐5p is overexpressed in bladder cancer and exerts pro‐oncogenic effects.^30^ However, miR‐516a‐5p is decreased in hepatocellular carcinoma^31^ ^,^ ^32^ and papillary thyroid cancer,^33^ and acknowledged as an anti‐oncogenic miRNA. In NSCLC, downregulated miR‐516a‐5p expression is clearly related with aggressive clinicopathological parameters.^34^ Furthermore, miR‐516a‐5p is an independent prognostic factor for predicting cancer relapse.^34^ Functionally, miR‐516a‐5p participates in the regulation of multiple malignant properties,^34^ which is consistent with our data. In mechanistic studies, FOXK1, a member of a subclass of Forkhead transcription factors, was a downstream target of miR‐516a‐5p in NSCLC. FOXK1 induces the Wnt/β‐catenin pathway and consequently regulates tumor genesis and progression.^18^ Here, our data further proved that the regulatory actions of RNASEH1‐AS1 on the Wnt/β‐catenin pathway were achieved by targeting miR‐516a‐5p/FOXK1. According to the subsequent rescue experiments, the miR‐516a‐5p/FOXK1 axis was further confirmed to be a downstream effector of the carcinostatic activities of RNASEH1‐AS1 in NSCLC cells. Overall, RNASEH1‐AS1 exacerbated the aggressiveness of NSCLC cells by controlling the miR‐516a‐5p/FOXK1/Wnt/β‐catenin pathway.

Our study had two limitations. First, we illustrated the detailed functions of RNASEH1‐AS1 in NSCLC and the downstream mechanisms; but, the underlying molecular events caused RNASEH1‐AS1 dysregulation were not unveiled. Second, the regulatory actions of RNASEH1‐AS1 on NSCLC cell metastasis in vivo were not explored. We will tackle the limitations in the near future.

## 5 CONCLUSION

The central observations of our research confirmed that high RNASEH1‐AS1 expression in NSCLC cells were related to poor patient prognosis. RNASEH1‐AS1 accelerated the malignant progression of NSCLC cells, and its effects on promoting NSCLC progression were achieved by sequestering miR‐516a‐5p and overexpressing FOXK1, which, in turn, activated the Wnt/β‐catenin pathway. Our newly identified RNASEH1‐AS1/miR‐516a‐5p/FOXK1/Wnt/β‐catenin network may offer an interesting foundation for NSCLC treatment in the clinic.

## CONFLICT OF INTEREST

No conflict of interest.

## AUTHOR CONTRIBUTIONS

Chan Zhang and Hui Ouyang designed the research. Chan Zhang, Jian Huang, and Ke Lou implemented all the experiments. Data analysis was performed by Hui Ouyang. Chan Zhang and Hui Ouyang drafted the manuscript.

## ETHICAL APPROVAL STATEMENT

The Research Ethics Committee of The Fourth Hospital of Changsha approved the research. The in vivo tumor xenograft experiments were implemented under the permission from the Animal Care and Use Committee of the Fourth Hospital of Changsha.

## Data Availability

The datasets used and/or analyzed during the present study are available from the corresponding author upon reasonable request.
